# Acute Unilateral Swelling of Parotid Gland After General Anesthesia: Anesthesia Mumps

**DOI:** 10.4274/balkanmedj.galenos.2019.2019.6.103

**Published:** 2019-10-28

**Authors:** Erhan Özyurt, Gülsüm Ekin Sarı, Selen Doğan

**Affiliations:** 1 Clinic of Anesthesiology and Reanimation, University of Health Sciences, Antalya Training and Research Hospital, Antalya, Turkey; 2 Clinic of Obstetrics and Gynecology, University of Health Sciences, Antalya Training and Research Hospital, Antalya, Turkey

To the Editor,

Anesthesia mumps is a unilateral or bilateral acute swelling of the parotid glands that develops after a surgery (1,2,3,4). It was first described in 1960, and the incidence is reported to be 0.16%-0.2% (5). However, the mechanisms that lead to anesthesia mumps have not yet been elucidated. We aimed to raise awareness of a rare case of anesthesia mumps, among both the anesthesia and the surgical teams.

Total abdominal hysterectomy and debulking surgery were planned for a 46-year-old female patient due to endometrium cancer. She did not have any co-morbid disease or abnormal laboratory findings before the surgery. In the operating room, after preoxygenation with 100% oxygen, we applied anesthesia induction with 0.2 mg/kg midazolam, 2.5 mg/kg propofol, 1 mcg/kg fentanyl, and 0.6 mg/kg rocuronium. We continued mask ventilation for 2 minutes with an average airway pressure of 20 cm H_2_O. After the endotracheal intubation, we used 50% oxygen and 50% air, 6 volume% desflurane, 0.5 mcg/kg/min remifentanil infusion, and 0.15 mg/kg rocronium for anesthesia maintenance. The operation was performed in supine position and lasted for 5 hours. During the operation, we administered 3,000 mL of crystalloid, 2 units of erythrocyte suspension, and 2 units of fresh frozen plasma to the patient. At the end of the operation, we extubated the patient using 2 mg/kg sugammadex and administered 1 g paracetamol and 100 mg tramadol for postoperative pain management. However, a sudden, painless swelling occurred in the right parotid region at the 5^th^ hour postoperatively. The patient was diagnosed with acute parotitis on physical examination and ultrasonography after an otorhinolaryngology consultation. We administered dexamethasone, non-steroid anti-inflammatory drugs, warm compression, and maintained hydration. The complaints regressed on the 11^th^ postoperative day, and the patient was discharged. An informed consent was obtained from the patient to publish data regarding her case.

Many factors are thought to play a role in the mechanism of anesthesia mumps (1,5). First, due to muscle relaxation during general anesthesia and positive pressure mask ventilation applied into the mouth, air enters the parotid glands retrogradely and causes pneumoparotitis (3). Additionally, anesthesia mumps may be the result of obstruction of the salivary canal in cases of excessive stress, coughing, and sneezing in patients in whom positive pressure ventilation is continued during awakening from general anesthesia (1). Another mechanism is the over-rotated head position and compression of the parotid gland, resulting in the obstruction of the parotid canal during prolonged operations. Moreover, drugs used during the operation, such as atropine, succinylcholine, morphine, and inhalation anesthesia, can lead to obstruction of the salivary tract in patients who do not achieve adequate hydration, by reducing and thickening salivary secretion (2). We assume this mechanism to be effective in our patient. Anesthesia mumps may regress spontaneously within 48 hours or there may be a need for a longer symptomatic treatment (2,3). Adequate hydration, pain control, and warm compress application can help relieve symptoms.

In conclusion, it is the most important issue to address the concerns of patients and their families by stating that anesthesia mumps is a temporary condition.
